# Preventive practices by households against Lassa fever transmission in Ondo State, Southwest, Nigeria

**DOI:** 10.11604/pamj.2022.43.176.32315

**Published:** 2022-12-05

**Authors:** Elvis Efe Isere, Akinola Ayoola Fatiregun, Nosa Eniye Omorogbe, Matthew Temitope Oluwole

**Affiliations:** 1 Department of Epidemiology and Medical Statistics, University of Ibadan, Ibadan, Nigeria,; 2 World Health Organization, Ondo State Field Office, Akure, Nigeria,; 3 Medical Laboratory Science Council of Nigeria, Abuja, Nigeria,; 4 Ondo State Ministry of Health, Akure, Nigeria

**Keywords:** Lassa fever, outbreak, preventive practice, risk communication, Household, Ondo State

## Abstract

**Introduction:**

continued transmission of Lassa fever has been recorded in 6 Local Government Areas (LGAs) of Ondo State in Southwest, Nigeria annually with high case fatality rates. Genomic analysis of the Lassa virus has indicated ongoing transmission from local rodents´ population to humans despite several public health interventions during the outbreak including risk communication on preventive practices against the disease. We assessed adherence to preventive practices by household against the spread of Lassa fever in these affected LGAs.

**Methods:**

a descriptive cross-sectional study was conducted among community members in the six affected Local Government Area (LGAs). A semi structured questionnaire was administered to 2992 consenting respondents to assess their reported preventive practice against Lassa fever while their observed practices was assessed using observation checklist. Data analysis was done using frequency, proportions, Chi-Square test and logistic regression of predictors of outcome variable with statistical significance set at p<0.05.

**Results:**

a higher proportion of the respondents were females (51.2%) compared to males (48.8%) with mean age of 43.04±13.97 years. Majority of the respondents (88.2%) were married with at least secondary education (76.7%). Majority of the respondents (80.2%) reported washing their hands with soap and water regularly, 84.6% washed their utensils before and after use likewise. However, 10.6% of the respondents reported not storing their food items in lid-covered containers while 61.9% practiced open air drying of food items by the roadside. Also, 34.3% of the respondents were observed to spread food items outside their home in the open air. Overall, 32.6% of the respondent were observed to have poor preventive practices against Lassa fever with their level of education as a significant determinant.

**Conclusion:**

the poor preventive practices observed among the respondents in this study could sustain the transmission of the virus hence there is also the need to further intensify enforcement of public health control measures for Lassa fever through existing community structures and institutions to stop the current and prevent future Lassa fever and other related outbreaks in the State.

## Introduction

Lassa fever (LF) is an acute viral haemorrhagic illness caused by the Lassa virus which is a single strangled RNA virus belonging to the Family Arenaviridae [1-5]. It is a zoonotic disease whose natural reservoir is a “Multimammate rat”, Mastomys natalensis. The Mastomys rats have been found to produce large number of offspring and are mostly found in most parts of Africa [5]. These rats live in and around most homes contaminating uncovered food items which serves as a medium for transmission of the disease to humans [5]. Primary mode of spread is from rodent to man through consumption of food item contaminated with the virus from excreta/urine of these rats, contact with the rodent excreta or urine in food or during hunting and processing of rats for consumption [6-9]. Also, the virus has the capacity for person-to-person spread, within households during care for sick relatives [6]. Community members who are at high risk of being infected with the disease are those living in rural areas and overcrowded environment with very poor sanitary conditions [5]. Transmission of the disease have also been reported among rural farmers who practice open air drying of their farm produce [5]. An outbreak of LF was confirmed in January 2016 in Ondo State, Southwest Nigeria [4] and span through 2017 to 2018 affecting mainly six Local Government Areas (LGAs) of the 18 LGAs in the State. Despite several public health interventions such as risk communication on several preventive practice against LF in various communities in the affected LGAs, continued transmission of the disease has persisted monthly with high case fatality rates recorded. Genomic analysis of the Lassa virus has indicated ongoing transmission from cross species of local rodents´ population to humans [10]. Hence, we assessed adherence of household preventive practice against the transmission of Lassa fever among community members in the affected areas to further guide future risk communication and public health interventions.

## Methods

### Study area and population

Nigeria is the most populous country in Africa, with an estimated population of over 160 million [11] and a growth rate of 3.8% per annum. Nigeria has six regional zones with varying ecologies, climates and population characteristics. The zones are divided into 36 States and the federal capital territory, which is further divided into 774 LGAs or districts and 8 812 administrative wards [12]. Ondo State is one of the 36 States in the Federal Republic of Nigeria situated between longitudes 40 151 E and 60 001E of the Greenwich meridian and latitudes 50 451N and 70 451 N, which are to the North of the equator, in the Southwestern geopolitical zone of the country [13]. The State has 18 LGAs with three senatorial districts; Ondo North, Central and South and a 2015 projected total population of about 4,489,756 based on the 2006 population census [13]. The climate of the areas is highly favoured for the agrarian activities and crops such as cocoa, kola nut, palm tree and arable crops like maize and tubers such as yam and cassava are grown annually [14]. The annual rainfall is between 1000mm and 1500mm with a high daily temperature of about 30^0^C. Most of the population consists of peasant farmers cultivating food and cash crops at a small-scale level. Hunters and livestock keeping is also a major occupation of the population of Ondo State who rear goats, sheep and also do some fish farming. Other economic activities in the State include trading and civil service [15]. The outbreak was restricted to six LGAs (Akure North, Akure South, Akoko Southwest, Ose, Owo and Idanre) in the north and central senatorial districts of the State. The study was conducted in six affected Lassa fever LGAs in Ondo State (Owo, Ose, Idanre, Akure South, Akure North, Akoko Southwest LGAs). The study population were members of community who resides permanently in selected communities within the LGAs.

**Sample size estimation:** the sample size was determined using the statistical formula for estimating the minimum sample size in descriptive health studies (n¼Z^2^pq/d^2^), where n¼ sample size, Z¼ standard normal deviate corresponding to confidence level at 95%¼ 1.96, d¼ degree of accuracy desire d¼= 0.05, p¼=49% have good practice of Lassa fever [16] while q¼=(1-p¼1-0.49¼=0.51); n¼1.962*0.49*0.51/(0.05)¼=384. Considering non responders we estimated a sample size of 422. Hence, the overall minimum sample size for the six LGA was obtained by multiplying the calculated sample size by 6 (2534 participants).

**Sampling method:** a multistage sampling technique was employed in this study. In stage 1, we selected 5 wards from the list wards in each Local Government Area (LGA) using simple random technique (balloting). In stage 2, 5 to 6 settlements out of an average of 30 settlements per ward were sampled by simple random technique (balloting). A total of 30 settlements were randomly selected from the list of settlements generated from each LGA. In stage 3, sampling of households from each selected settlement was done using systematic sampling technique. Finally in stage 4, heads of households or assigned adults older than 18 years in selected households were interviewed.

**Data collection instrument for the study:** the questionnaire was adapted from previous studies on preventive practices against Lassa fever transmission in other parts of Nigeria [7,17] and translated from English to local languages. The questionnaire had four sections: section A addressed Socio-demographics of respondents such as age, gender, religion, marital status while section B assessed the housing quality and hygiene of respondents, section C assessed the respondent´s reported preventive practices and section D assessed the respondent´s observed preventive practices.

**Study design and data collection:** a descriptive cross-sectional community-based survey was used. A semi-structured questionnaire was administered to participants by trained research assistants using Open Data Kit (ODK) application. The questionnaire captured information on socio-demographic variables, housing quality and hygiene (16 variables) and preventive practice against Lassa fever (16 variables) was used to assess reported practices. Also, an observation checklist (16 variables) was used to assess the standard of living and observed practice by each respondent interviewed.

**Data analysis:** the data obtained were analyzed using frequency counts and percentages using IBM SPSS version 20.0 statistical software. Overall practice score was calculated by assigning a value of 1 to every right preventive practice variable under practice sections and “0” for wrong preventive practice among the respondents with the average practice score calculated from the total score. A total practice assessment score of 16, with range 0-7 score considered bad practice while 8-16 was considered good practice. Also, a total observed practice score of 16, with range 0-7 was considered bad practice while 8-16 was considered good practice likewise. Bivariate chi-square test and multivariate logistic analyses were conducted on respondents´ socio-demographic characteristics and preventive practices against Lassa fever. Variables in the bivariate test with p value of < 0.2 were included in the multivariate model. A p value < 0.05 will be accepted as significant.

**Ethical consideration:** the study was conducted as part of an outbreak control investigation hence ethical approval was obtained from the Ondo State Health Research Ethics Committee of the Ondo State Ministry of Health, Akure, Nigeria with protocol number OSHREC11/10/22/480. Also, Informed consent was obtained from the respondents. They were made to understand that participation is voluntary and there was no consequence for non-participation. All information obtained was kept confidential.

## Results

Table 1 shows the socio-demographic distribution of the respondents. The mean age was 43.04±13.97 years and more than half (51.2%) were female. Most of the respondents were married (88.2%), of Christian faith (82.3%) and of Yoruba extraction (81.8%). Table 2 shows the reported practices by respondents with 27.3% of respondents indicating that their families did engage in catching or handling rats and 26.4% have not taken preventive action to protect their family and community from Lassa fever. The unavailability of dumbing sites was reported by 36.7% while 38.4% reported having alternative means of spreading food items apart from outside. Most of the respondents (70.2%) have a toilet facility, while 79.5% have a clean water source. Also, most of the respondents (80.2%) reported washing their hands with soap and water regularly and 84.6% washed utensils immediately before and after use. Some respondents (38.1%) do not spread food items by the roadside or in an open place and 10.6% reported they do not store food items in a lid-covered container while 17.7% reported they do not touch a life or dead rats with bare hands. Majority of the respondents (95.6%) reportedly cooked all foods properly before eating while 92.4% wash fruits before eating with clean water. More than half of the respondents 63.5% claimed they blocked all rodents´ holes in the house while 53.6% disposed of their waste in a covered dustbin. On the categorization of reported practices, only 19% of respondents reported poor practices.

**Table 1:** socio-demographic characteristics of the respondents among households in Ondo State

Variables	Frequency (N=2992)	Percentage (%)
LGAs		
Akoko South West	503	16.8
Akure North	501	16.7
Akure South	479	16
Idanre	482	16.1
Ose	485	16.2
Owo	542	18.1
Gender		
Female	2531	51.2
Male	1461	48.8
**Age**		
<26	268	9
26-35	710	23.7
≥36	2014	67.3
**Religion**		
Christianity	2461	82.3
Islam	481	16.2
Other	12	0.4
Traditional	34	1.1
**Occupation**		
Civil servant	348	11.6
Clergy	124	4.1
Other including farmers	2296	76.7
Student	224	7.5
**Ethnicity**		
Hausa	32	1.1
Igbo	225	7.5
Yoruba	2446	81.8
Other	289	9.7
Marital status		
Divorced	19	0.6
Married	2578	88.2
Single	299	10
Widowed	2992	3.2
**Educational level**		
Primary	2098	36.7
Secondary	2298	40
Tertiary	696	23.3

**Table 2:** preventive practice among households in Ondo State

Variables	Frequency (N=2992)	Percentage (%)
Does any member of your family engage in caching or handling rats?		
Yes	816	27.3
No	2176	72.7
Since the time you heard about Lassa fever, have you taken any preventive action to protect yourself, your family and community from the disease?		
Yes	2203	73.6
No	789	26.4
Is a dumping site available?		
Yes	1699	36.7
No	1699	36.7
Do you have alternative means of spreading food items apart from outside?		
Yes	1149	38.4
No	2101	61.6
Do you have toilet?		
Yes	2101	70.2
No	891	29.8
Do you have water source?		
Yes	2379	79.5
No	613	20.5
Washing hands with soap and water regularly		
Yes	2401	80.2
No	591	19.8
Wash utensils immediately before and after use		
Yes	2532	84.6
No	460	15.4
Don’t spread food items by the road side or in an open place		
Yes	1141	38.1
No	1851	61.9
Store food item in a lid-covered container		
Yes	2675	89.4
No	317	10.6
Don’t touch life or dead rat with bear hands		
Yes	530	17.7
No	2462	82.3
Cooked all foods properly before eating		
Yes	2861	95.6
No	131	4.4
wash fruits before eating with clean water		
Yes	2766	92.4
No	226	7.6
Block all rodents’ holes in the house		
Yes	1900	63.5
No	1092	36.5
Dispose waste in a covered dustbin		
Yes	1603	53.6
No	1389	46.4
Don’t touch sick or dead person with bear hands		
Yes	644	21.5
No	2348	78.5

Results from Table 3 show that 41.2% lived in a building made up of mud, while 35.5% of the respondent had the presence of rodent burrows on the wall of their houses with rodent footprints, scratches, marks and tracks. Almost three-fourths (73.3%) of the respondents had open space on the roof, and 73.3% had a house with a well-sealed door. The majority (76.6%) had windows properly sealed or well protected while 46.2% had extensive vegetation around the house or on the house. About one-fourth of the respondents (24.7%) had debris around the house and 21.7% had sewage around the house untidy. Only 10.9% do not have storage of water in a covered container and 31.0% had waste collection point very untidy and close to the house. About one-fourth of the respondent had pit toilet very close to the house and 11.4% had septic tank uncovered or partially covered. Domestic animals were kept by 67.4% while it was observed that 27.2% had hand washing basin and 86.5% had food items properly protected. Moreover, 34.3% of the respondents were observed to spread food items outside. On the categorization of the observed practices, 32.6% of households were estimated to have poor practices. Table 4 shows statistically significant associations between the observed housing and household hygiene practices and the LGAs location (Ose LGA), gender, age, religion, ethnicity, educational level and knowledge of Lass fever.

**Table 3:** housing quality and hygiene among households in Ondo State

Variables	Frequency (N=2992)	Percentage (%)
Building material made up of mud		
Yes	1232	41.2
No	1656	55.3
Not applicable	104	3.5
Presence of rodent burrows on the wall of the house with rodent footprint, scratches, marks and trait		
Yes	1063	35.5
No	1775	59.3
Not applicable	154	5.2
Open space on the roof		
Yes	2192	73.3
No	699	23.4
Not applicable	109	3.4
Does house have a well-sealed door		
Yes	2192	73.3
No	699	23.4
Not applicable	109	3.4
Windows properly sealed or well protected		
Yes	2291	76.6
No	590	19.7
Not applicable	102	3.7
Extensive vegetation around house or on the house		
Yes	1381	46.2
No	1502	50.2
Not applicable	109	3.7
Food debris around the house		
Yes	739	24.7
No	2106	70.4
Not applicable	127	4.9
Sewage around the house untidy		
Yes	650	21.7
No	2126	71.1
Not applicable	216	7.2
Storage of water in covered container		
Yes	2503	83.7
No	326	10.9
Not applicable	163	5.5
Waste collection point very untidy and close to the house		
Yes	927	31
No	1947	65.1
Not applicable	118	4
Pit toilet facility very close to the house		
Yes	724	24.2
No	1814	60.6
Not applicable	454	15.2
Septic tank uncovered or partially covered		
Yes	341	11.4
No	2003	66.9
Not applicable	648	207
Domestic animals around the house		
Yes	2018	67.4
No	859	28.7
Not applicable	115	3.9
Presence of a handwash basin		
Yes	813	27.2
No	1837	61.4
Not applicable	352	11.5
Are Food items properly protected		
Yes	2587	86.5
No	250	8.4
Not applicable	152	5.2
Food items properly spread outside		
Yes	1025	34.3
No	1852	61.9
Not applicable	115	3.9

**Table 4:** association between respondent’s sociodemographic characteristics and observed preventive practice in ondo state

	Practice Score				
	Good	Bad	Total	Chi-Square	P-Value
	n(%)	n(%)	n(%)		
LGAs					
Akoko southwest	360(71.6)	143(28.4)	503(100.0)	14.826	0.011
Akure North	320(63.9)	181(36.1)	501(100.0)		
Akure South	333(69.5)	146(30.5)	479(100.0)		
Idanre	317(65.8)	165(34.2)	482(100.0)		
Ose	306(63.1)	179(36.9)	485(100.0)		
Owo	382(70.5)	160(29.5)	542(100.0)		
**Gender**					
Female	1055(68.9)	476(31.1)	1531(100.0)	3.055	0.080
Male	963(65.9)	498(34.1)	1461(100.0)		
**Age**					
<26*	170(63.4)	98(36.6)	268(100.0)	3.701	0.157
26-35	495(69.7)	215(30.3)	710(100.0)		
≥36	1353(67.2)	661(32.8)	2014(100.0)		
**Religion**					
Christianity	1687(68.5)	774(31.5)	2461(100.0)	11.973	0.070
Islam	301(62.1)	184(37.9)	485(100.0)		
Traditional	5(41.7)	7(58.3)	34(100.0)		
Others	25(73.5)	9(26.5)	12(100.0)		
**Occupation**					
Civil servant	298(85.6)	50(14.4)	348(100.0)	72.978	0.000
Clergy	94(75.8)	30(24.2)	124(100.0)		
Student	1464(72.3)	832(27.7)	2296(100.0)		
Others including farmers	162(63.8)	62(36.2)	224(100.0)		
**Ethnicity**					
Hausa	19(59.4)	13(40.6)	32(100.0)	1.028	0.000
Igbo	129(57.3)	96(42.7)	225(100.0)		
Yoruba	1744(43.6)	702(56.4)	2446(100.0)		
Others	126(71.3)	163(28.7)	289(100.0)		
**Marital status**					
Divorced	11(57.9)	8(42.1)	19(100.0)	1.374	0.712
Married	1737(67.4)	841(32.6)	2578(100.0)		
Single	207(69.3)	92(30.8)	299(100.0)		
Widowed	63(65.6)	33(34.4)	96(100.0)		
**Educational level**					
Primary	620(56.5)	478(43.5)	1098(100.0)	1.449	0.000
Secondary	815(68.0)	383(32.0)	1198(100.0)		
Tertiary	583(83.8)	113(16.2)	696(100.0)		

## Discussion

Primary transmission of Lassa fever is known to occur in communities with poor sanitation or crowded living conditions [3]. In this report, we present the results of the assessment of the reported preventive practices and observed community hygiene among households in communities in Ondo State where at least a suspected case of Lassa fever or contact has been reported in an outbreak in early 2018. A total of 2992 households in 180 communities spanning 6 out the 18 LGAs in the State was covered, making it the largest survey on Lassa fever in the State or that we were able to identify in the literature. A handful of the respondent family members still engaged in practices that promotes transmission of the disease such as, catching or handling of rats, not taken preventive action to protect themselves, no available dumping site and no alternative means of spreading food items apart from outside, no toilet facilities, no clean water source, spreading of food items by the roadside, food not stored in lid covered container and touching life or dead rat with bared hands among others as reported. On observation of housing quality and household hygiene, a third of the respondents´ households were categorized as poor with the building materials made up of mud, had the presence of rodent burrows on the wall of the house with rodent footprints, scratches and marks. Almost three-fourths of the respondents had open space on the roof and about a third of houses had no well-sealed door. Almost half had extensive vegetation around the house or on the house and one-fourth of the respondent had waste debris around the house. A fifth of the respondent had sewage around the house untidy. Few respondents´ households had a waste collection point very untidy and close to the house. And the majority had domestic animals around the house. Although the majority reported having a toilet facility and clean water source only a few had handwashing basin. The predictors of housing quality and household hygiene were LGA location, ethnic persuasion, educational level, gender and age.

The primary transmission of Lassa fever has been found to be the main mode in our environment in a recent report [4]. This transmission occurs through direct exposure to rodents´ fluids such as urine, saliva and blood or indirectly through exposure via surfaces and foodstuffs contaminated by these fluids [18]. Our findings suggesting that a handful of our respondents had interaction or engaged in catching or handling rats are therefore practices likely promoting the spread of the virus in the endemic communities. In studies of Lassa fever in Sierra Leone, the reservoir of Lassa virus constituted 50%-60% of the rodents captured in houses but only 10%-20% of those captured in surrounding agriculture and bush areas, a finding suggesting that houses are the most important location for transmission of Lassa virus [19]. More so, recent evidence reveals that apart from the multimammate rats, Mastomys natalensis, other rodent reservoirs have been identified, though their contribution to human infection is still unknown [20].

Housing characteristics that lead to an increased rodent density in and around households are likely risk factors for Lassa fever transmission to humans [18]. A third of the houses surveyed are categorized as poor with building materials made up of mud, the presence of burrows on the walls and rodent footpaths, scratches and marks. Almost three-fourths of the respondent had open space on the roof and about a third of houses had no well-sealed door. Almost half had extensive vegetation around the house or on the house and one-fourth of the respondent had waste debris around the house. These are factors that could promote rodents´ infestations. Although there is little information describing the specification for barriers for rodent human interaction such as construction method and domestic organization, a study found that most thatched houses with mud and wattle walls and earth floor provide opportunities for burrows by rodents, thereby increasing rodents´ density [18]. In addition, a study in Sao Paulo, Brazil found that the main environmental factors leading to rodent infestation of home and premises are access by sewage system, building structure, harbourage by ceiling cracks, wall cracks, waste material, building material, bushes and food sources around premises [21]. Similarly, a study in Lao revealed that trophic factors such as exposed food (indoors) and garbage (outdoors), and structural features such as open ceilings (indoors) and rat harbourage in gardens (outdoors) ranked highest as explanatory variables for indoor and outdoor rodent infestations [22].

However, interventions on socio-economic conditions have been reported to be very difficult to accomplish and they depend almost exclusively on public policies, so the results of such actions will be obtained in a long-term [21]. It has been suggested that basic sanitation measures (e.g. removing garbage), rodent proofing (e.g. for stored food) and simple rodent control by using traps inside houses could reduce rodent infestations levels cheaply [22]. There are, however, some risks of transmission of Lassa fever in handling rats caught by traps, hence proper infection prevention measures need to be put in place to prevent transmission of diseases including Lassa fever using such method. Our findings underline the need for an appropriate rodent management strategy to reduce rodent infestation of homes. The role of domestic animals in the transmission and prevention of Lassa fever is unknown. Studies have found other rats harbouring the Lassa fever virus, though their role in transmission to humans is understudied [20]. Cat, a predator of rats, has been suggested to be kept at homes, to keep rats away, but the effectiveness of such measure and potential for cats to transmit the infection have not been determined.

With regards to predictors of community and household hygiene, a study in rural India described two types of drivers; structural and psychosocial [23]. The structural factors include socioeconomic and ecologic characteristics, while psychosocial drivers include perceived unaffordability of measures such as toilet facility, hygiene and sanitation knowledge, perceived health risks, perceived benefits of ownership and use of toilets and perceived descriptive social norms. In this study, we found that respondents´ location, ethnic persuasion, educational level, gender and age were predictors of household hygiene. Those household heads with tertiary education, who are females and older tend to have better hygiene practices. Similarly, the study in India found structural constraints related to education, economic and social inequality predicting toilet ownership [23]. It was suggested that persuasive strategies that manipulate social norms around sanitation, particularly if they simultaneously address perceptions around the financial unaffordability of toilets and around the benefits of toilets have the potential in improving community hygiene.

**Limitation:** the limitation of our study is well recognized. A cross-sectional descriptive household survey with potential for recall and social desirability biases. We incorporated an observational environmental assessment checklist into the data collection tool to provide observed data for triangulation with the reported data by respondents. The dissimilar findings from the observed and reported poor practices of 32.6% and 19.0% respectively could be partly contributed to by recall or social desirability bias. Although the two tools do not have the same questions, the construct is similar, and we assumed that the observed practice´s data were more reliable. Our findings could also have been influenced by the timing of the study, which was towards the end of an outbreak in the State and the country. There were ongoing sensitizations on mass media and community engagements during the data collection period which might have influenced the results.

## Conclusion

Despite several risk communication interventions implemented, a handful of our respondents´ preventive and hygiene practices were poor and could sustain the transmission of the virus, hence the need to target sensitization and community engagements in the groups identified in this study. There is also the need to further intensify enforcement of public health control measures for Lassa fever through existing community structures and institutions to stop the current and prevent future Lassa fever and other related outbreaks in the State.

### What is known about this topic

- *Lassa fever is endemic in Ondo State, Nigeria with a high annual case fatality rate;*
- *Sustained transmission of Lassa fever has continued in Ondo State yearly despite several risk communication and preventive measures rolled out by government public health authorities*.

### What this study adds

- *Poor preventive practices among households within communities are key drivers of the annual Lassa fever transmission and outbreaks recorded in Ondo State, Nigeria;*
- *Community interventions targeting gaps in preventive practices of households towards Lassa fever control would address the menace of Lassa fever recorded yearly in Ondo State, Nigeria*.
